# Receptors for short-chain fatty acids in brush cells at the “gastric groove”

**DOI:** 10.3389/fphys.2014.00152

**Published:** 2014-04-16

**Authors:** Julia Anna-Maria Eberle, Patricia Widmayer, Heinz Breer

**Affiliations:** Institute of Physiology, University of HohenheimStuttgart, Germany

**Keywords:** short-chain fatty acid receptors, GPR41, GPR43, brush cells, “limiting ridge”, forestomach

## Abstract

In the stomach of rodents clusters of brush cells are arranged at the “gastric groove,” immediately at the transition zone from the non-glandular reservoir compartment to the glandular digestive compartment. Based on their taste cell-like molecular phenotype it has been speculated that the cells may be capable to sense constituents of the ingested food, however, searches for nutrient receptors have not been successful. In this study, it was hypothesized that the cells may express receptors for short-chain fatty acids, metabolites generated by microorganisms during the storage of ingested food in the murine forestomach, which lacks the acidic milieu of more posterior regions of the stomach and is colonized with numerous microbiota. Experimental approaches, including RT-PCR analysis and immunohistochemical studies, revealed that the majority of these brush cells express the G-protein coupled receptor types GPR41 (FFAR3) and GPR43 (FFAR2), which are activated by short-chain fatty acids. Both, the GPR41 receptor proteins as well as an appropriate G-protein, α-gustducin, were found to be segregated at the apical brush border of the cells, indicating a direct contact with the luminal content of this gastric region. The exposure of microvillar processes with appropriate receptors and signaling elements to the gastric lumen suggests that the brush cells may in fact be capable to sense the short-chain fatty acids which originate from fermentation processes during the retention of ingested food in the anterior part of the stomach.

## Introduction

The stomach of rodents comprises two well-defined areas, a thin-walled, non-glandular compartment, the forestomach (fundus), which serves as a reservoir for food and the thick-walled, glandular section, the corpus, where secretory glands produce digestive enzymes and where the actual digestive processes take place (Frantz et al., 1991; Matsukura and Asano, 1997). How the transfer of the ingested food from the anterior storage compartment into the posterior digestive compartment is regulated remains unclear. Previous studies have shown that the two sections are separated by an anatomical structure, the limiting ridge or margo plicatus (Wattel and Geuze, 1978; Luciano and Reale, 1992). Recent cellular analysis of this structure revealed that brush cells beneath the “limiting ridge” may be sensory cells. These cells, which are arranged in a palisade-like manner forming a band which borders the whole length of the corpus epithelium, express important signal transduction elements, such as gustducin, PLCβ2 and TRPM5, typical for gustatory sensory “type II” cells (Höfer et al., 1996; Hass et al., 2007; Eberle et al., 2013). It has therefore been proposed that they may be capable of sensing nutrient or non-nutrient constituents of the ingested food. However, so far appropriate receptors for recognizing food constituents have not been identified. Comparative studies have shown that in most animals, especially in rodents, the anterior compartment of the stomach harbors a much larger population of microorganisms than the posterior compartment and that the pH of the chyme in the section is sufficiently high to permit bacterial multiplication (Smith, 1965). It is thus conceivable that during the storage period of the ingested food active fermentation processes occur and that microflora-derived metabolic products are generated. Due to the mainly anaerobic metabolism the main metabolic end-products are presumably short-chain fatty acids (SCFAs), such as acetic, proprionic and butyric acid (den Besten et al., 2013). In the colon SCFAs are not only readily absorbed and used as nutrients in the intestine or enter the peripheral circulation (Wong et al., 2006) but also influence key functions of the gastrointestinal tract (Cummings et al., 1995). The concept that short-chain fatty acids may be involved in signaling processes was strongly supported by the discovery of G-protein coupled receptors which are activated by free fatty acids. Of particular interest are the receptor types GPR41 (FFAR3) and GPR43 (FFAR2) which respond to SCFAs, especially acetate, propionate, and butyrate (Brown et al., 2003; Le Poul et al., 2003; Nilsson et al., 2003). Moreover, evidence emerged indicating that the short-chain fatty acid receptor, GPR43, is expressed in the intestinal mucosa (Karaki et al., 2006, 2008). Therefore, in this study we set out to explore whether receptors for short-chain fatty acids are expressed at the “limiting ridge” and whether they occur in the candidate sensory cells at the “gastric groove.”

## Material and methods

### Mice

Analyses were performed with wild type mouse strains C57/BL6J purchased from Charles River (Sulzfeld, Germany). Animals were fed with standard laboratory chow *ad libitum* and had free access to water. All experiments comply with the Principles of animal care, publication no. 85–23, revised 1985, of the National Institutes of Health and with the current laws of Germany. For tissue preparations, prior to perfusion animals were killed by inhalation of lethal doses of carbon dioxide delivered by a compressed gas cylinder.

### RNA isolation and cDNA synthesis

Total RNA was isolated from dissected tissue preparations of the stomach compartments with a Nucleo Spin RNA kit (Macherey-Nagel, Düren, Germany) according to the manufacturer's protocol. To ensure the complete removal of DNA, a DNase digestion (DNaseI, LifeTechnologies, Carlsbad, CA, USA) step was included. Subsequently, 1.0 μ g total RNA was reverse transcribed using oligo (dT) primers and SuperScript III Reverse Transcriptase (RT; Invitrogen, Carlsbad, CA, USA). RNA integrity of each sample was confirmed by the amplification of the housekeeping gene for the ribosomal protein L8 (RpL8) with intron spanning primers to verify the DNA removal.

### Reverse transcriptase polymerase chain reaction (RT-PCR)

RT-PCR amplification was conducted by using normalized cDNA from different tissues of the stomach compartments. PCR amplifications were performed with the following primer combinations:

GPR41 forward, 5′- CAG AGT GCC AGT TGT CCA ATA-3′; GPR41 reverse, 5′-ATG CCA GGA ACC AAC AGA CT-3′; GPR43 forward, 5′-CAA ACT CGG GAT GCT TCA G-3′; GPR43 reverse, 5′-AGC AGC AAC AGG AGC AAG TC-3′.

RT-PCR was carried out using High Fidelity PCR Enzyme Mix (Fermentas, St. Leon-Rot, Germany) and a Peltier PTC-200 thermo cycler (MJ Research). For amplification the following PCR cycling profiles were used with annealing temperatures adjusted to the used primer combinations and optimized numbers of amplification cycles, as specified in the following.

For GPR41:

One cycle: 4 min at 94°C; 20 cycles: 30s at 94°C, 30 s at 65°C with −0.5°C per cycle, 40 s at 72°C; 25 cycles: 30 s at 94°C, 30 s at 55°C, 40 s at 72°C; and one cycle: 3 min at 72°C.

For GPR43:

One cycle: 4min at 94°C; 8 cycles: 30 s at 94°C, 30 s at 68°C with −0.5°C per cycle, 40 s at 72°C; 25 cycles: 30 s at 94°C, 30 s at 64°C, 40 s at 72°C; and one cycle: 3 min at 72°C.

PCR products were run on 1.5% agarose gels containing EtdBr. Amplification of a 205 bp fragment from mouse housekeeping control gene ribosomal protein l8 (rpl8) was used as control to confirm equal quality and quantity of the cDNA preparations.

### Tissue preparation

For immunohistochemistry, stomachs of adult mice were dissected in 1 × PBS and fixed as described below.

For immunoreactivity to GPR41, CK18, TRPM5, and gustducin antibodies mice were gassed with CO_2_ and perfused via the left heart ventricle with 1 × PBS followed by 4% ice-cold paraformaldehyde with 0.1% glutardialdehyde (in 150 mm phosphate buffer, pH 7.4). After perfusion the tissue was fixed in 1:1 4% PFA:1 × PBS for 24 h.

For double-labeling experiments with GPR43 and TRPM5 antibodies, the stomach was fixed in 4% paraformaldehyde (in 150 mM phosphate buffer, pH 7.4) for 7 h at 4°C.

After fixation the tissue was cryoprotected by incubation in 25% sucrose overnight at 4°C. Finally, the tissue was embedded in Tissue Freezing Medium and quickly frozen on liquid nitrogen. Cryosections (6 μm) were generated using a CM3050S cryostat (Leica Microsystems, Bensheim, Germany) and adhered to Superfrost Plus microscope slides (Menzel Gläser, Braunschweig, Germany).

### Immunohistochemistry

Cryosections were air-dried, rinsed in 1 × PBS for 10 min at room temperature and blocked in 0.3% Triton X-100 in 1 × PBS containing either 10% normal goat serum (NGS; Dianova, Hamburg, Germany) or 10% normal donkey serum (NDS; Dianova, Hamburg, Germany) for 30 min at room temperature. For immunostaining with CK18-antibody and in part for GPR41-antibody cryosections underwent citrate-antigen-retrieval. Therefore, frozen sections were incubated in sodium citrate buffer (10 mM sodium citrate, 0.05% Tween 20, pH 6.0) for 45 min at 4°C. Afterwards sections were immersed in the same sodium citrate buffer for 1–5 min at 100°C. After three rinses for 5 min in 1× PBS, cryosections were blocked in 0.3% Triton X-100 in 1 × PBS containing either 10% normal goat serum (NGS) or 10% normal donkey serum (NDS) for 30 min at room temperature. For single- and double-labeling experiments, primary antibodies were diluted in 0.3% Triton X-100 in 1 × PBS containing either 10% NGS or 10% NDS. Antibodies were used in the following dilutions: rabbit-anti-GPR41 (sc-98332, Santa Cruz Biotechnology, SantaCruz, CA, USA) 1:30; goat-anti-GPR41 (sc-131166, Santa Cruz Biotechnology, SantaCruz, CA, USA) 1:30; goat-anti-GPR43 (sc-28424, Santa Cruz Biotechnology, SantaCruz, CA, USA) mouse anti-cytokeratin18 (61028; Progen Biotechnik, Heidelberg, Germany) 1:80; rabbit anti-TRPM5 serum73 [purified antibody (AB-321) described in Kaske et al. (2007)] 1:1000; rabbit anti- gustducin [described in Sothilingam et al. (2011)] 1:150.

Blocked sections were incubated with the diluted primary antibodies overnight at 4°C. After washing in 1 × PBS, the bound primary antibodies were visualized using appropriate secondary antibodies conjugated to Alexa 488 or Alexa 568 (Invitrogen, Karlsruhe, Germany, 1:500) diluted in 1 × PBS with 0.3% Triton X-100 containing either 10% NGS or 10% NDS for 2 h at room temperature. After three rinses for 5 min in 1 × PBS, the sections were counterstained with 4,6-diamidino-2-phenylindole (DAPI; lμg/mL, Sigma Aldrich, Schnelldorf, Germany) for 3 min at room temperature, rinsed with bidest and finally mounted in MOWIOL. No immunoreactivity could be observed when the primary antibodies were omitted.

### Microscopy and photography

Immunohistochemical staining was documented by using a Zeiss Axiophot microscope (Carl Zeiss MicroImaging, Jena, Germany). Images were captured using a “Sensi-Cam” CCD-camera (PCOimaging, Kelheim, Germany). Images were adjusted for contrast in AxioVision LE Rel. 4.3 (Carl Zeiss MicroImaging, Jena, Germany) and arranged in PowerPoint (Microsoft).

## Results

**Figure 1:**
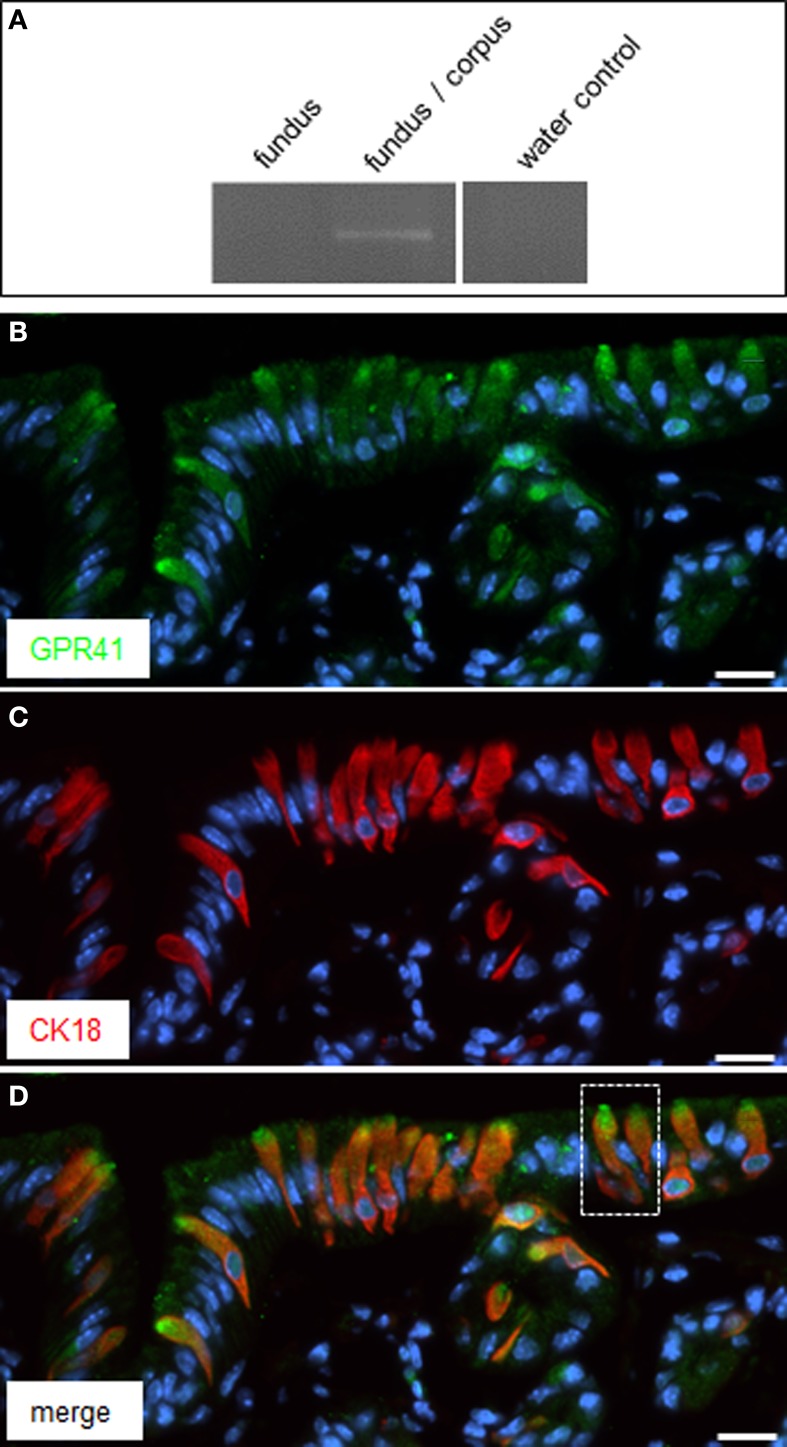
**Expression of GPR41 in CK18 positive brush cells at the “gastric groove.” (A)** Reverse transcription polymerase chain reaction (RT-PCR) experiments were performed with primer pairs specific for GPR41 (455 bp). Normalized cDNA from the fundus and fundus/corpus transition zone, respectively, was analyzed; for the fundus/corpus transition zone an amplicon of the expected size was obtained. Water control without template showed no amplicon. **(B)** Immunostaining of tissue sections of the corporal mucosa region underneath the “limiting ridge” with an antibody against GPR41. Several cells exhibited immunoreactivity *(green*) most prominent at their apical pole. **(C)** The CK18-antibody revealed a staining pattern *(red*) reminiscent of that in **(B**) except staining of the most apical cell pole. **(D)** Overlay of **(B)** and **(C)** clearly demonstrated co-expression of GPR41 and CK18 in the same population of cells but different subcellular staining patterns for both antibodies. Magnification of the *boxed area* is shown in Figures 2A–C. Sections were counterstained with DAPI (*blue*). *Scale bars*: **(B–D)** = 20 μm.

**Figure 2:**
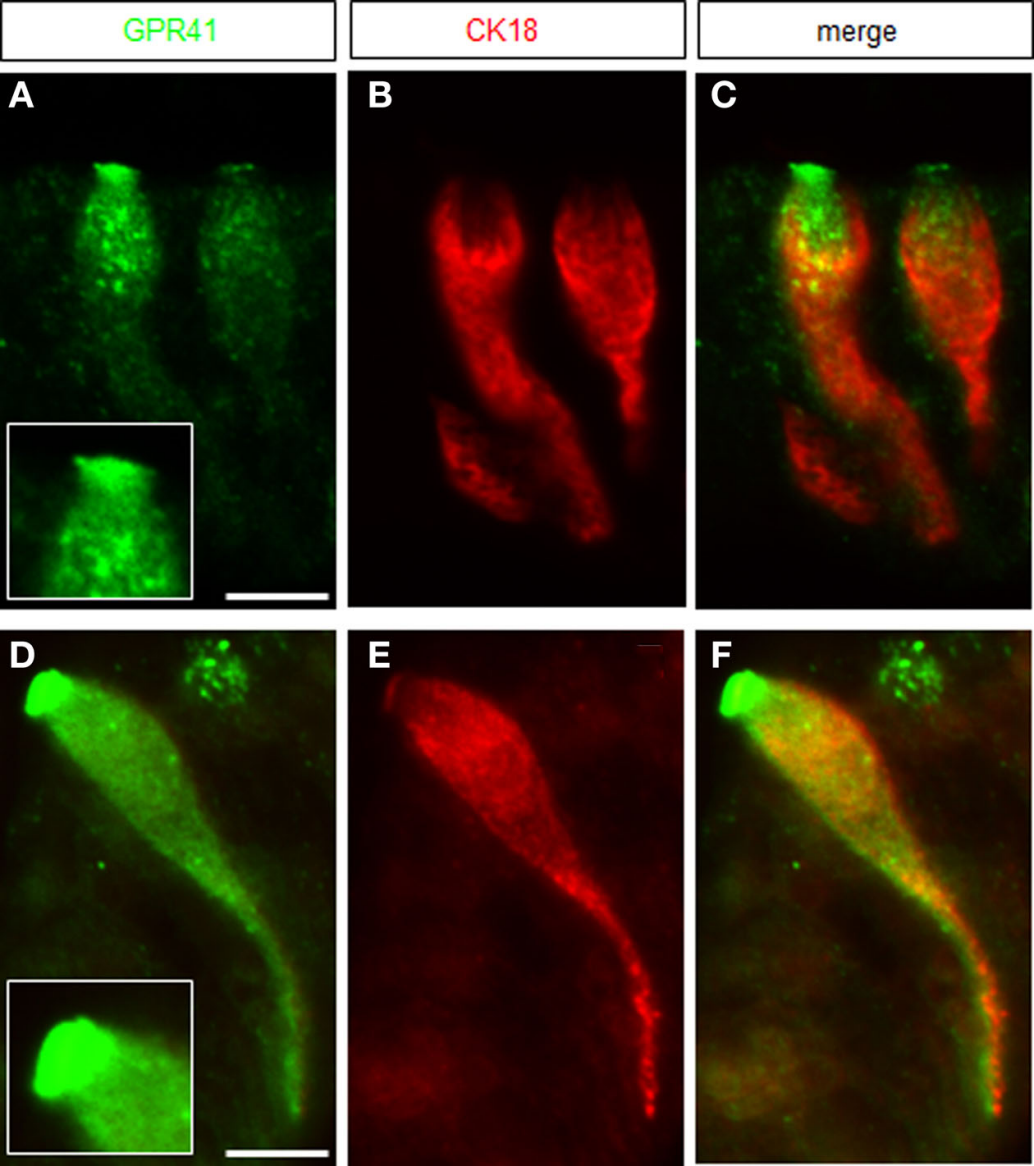
**Magnification of GPR41 immunoreactive brush cells. (A)** GPR41 immunostaining *(green)* was most intense at the apical cell pole revealing a dense brush of microvilli, enlarged in the *inset boxed area*. **(B)** Immunostaining for CK18-antibody resulted in strong labeling of the cytoplasm exhibiting a different staining pattern than the GPR41-antibody. **(C)** Overlay of **(A)** and **(B)** pointed out the differing staining patterns of both antibodies. **(D)** Immunoreactivity for GPR41-antibody *(green)* labeled the entire cell body with the most intense staining of the apical microvilli. Notable was the long basolateral process of the cell. **(E)** Immunofluorescence for CK18 was mainly concentrated in the cytoplasm of the cell. **(F)** Overlay of **(D)** and **(E)** demonstrated colocalization of GPR41 and CK18 immunostaining in the cytoplasm including the long basolateral process whereas apical microvilli only were strongly stained by the GPR41-antibody. *Scale bars*: **(A–F)** = 10 μm.

In the region of transition between the keratinized fundus mucosa and the glandular corpus mucosa there are numerous clusters of brush cells which are arranged in a palisade-like manner (Eberle et al., 2013). These brush cells are considered as candidate chemosensory cells (Höfer et al., 1996; Hass et al., 2007, 2010; Eberle et al., 2013). However, the functional implications of the chemosensory capacity of these cells are completely elusive; furthermore, it is unclear whether the cells do in fact sense any of the food ingredients. In view of the fermentation processes in the anterior part of the stomach, it seems a possibility that brush cells at the fundus/corpus border may be tuned to register microbiota-derived metabolites, such as short-chain fatty acids (SCFAs). As a first site step to scrutinize this concept, tissue samples from the “limiting ridge” area of the stomach were assessed for a possible expression of receptors for SCFAs. A promising candidate is the receptortype GPR41/FFAR3, a G-protein coupled receptor which has previously been shown to be activated by SCFAs (Brown et al., 2003; Le Poul et al., 2003). Therefore, RT-PCR analyses were conducted with tissue samples from at least three animals with primer pairs specific for GPR41/FFAR3. A distinct band was amplified from cDNA of the transition zone between fundus and corpus (Figure 1A); no amplification product was obtained with cDNA from the fundus compartment. In order to elucidate which cell type at the “limiting ridge” region expresses the GPR41 receptor, immunohistochemical experiments were performed using a specific antibody for GPR41. A typical result is depicted in Figure 1B, showing a variety of immunoreactive cells. The labeled cells are arranged at the distal wall of the “gastric groove” below the “limiting ridge.” To elucidate whether the immunoreactive cells belong to the brush cell population, which accounts for approximately 30% of the cells in this region (Akimori et al., 2011; Eberle et al., 2013), double-labeling approaches were performed using an antibody against CK18, a marker for brush cells (Höfer and Drenckhahn, 1996). As shown in Figure 1C several strongly stained elongate CK18 positive cells were visible at the “gastric groove.” An overlay of immunostainings for the two antibodies (Figure 1D) clearly demonstrated that all the GPR41 positive cells also express CK18 and hence are part of the brush cell population. Although the antibodies for GPR41 and for CK18 stained the same cell population, the subcellular staining pattern was different. While the immunoreactivity for CK18 resulted in a strong cytoplasmic labeling including the apical and basal processes, the immunoreactivity for GPR41 was particular strong at the most apical part of the cells. This differential staining pattern is more explicitly illustrated in Figure 2. Antibodies for GPR41 (Figures 2A,D) strongly labeled the apical region, probably the brush of microvilli (magnified in the inset *boxed area*) while the cytoplasm exhibited fainter fluorescence. By contrast, CK18 immunofluorescence (Figures 2B,E) was distributed throughout the cytoplasm but was excluded from the most apical cell region; this becomes even more clear in the overlay of both immunostainings (Figures 2C,F).

**Figure 3:**
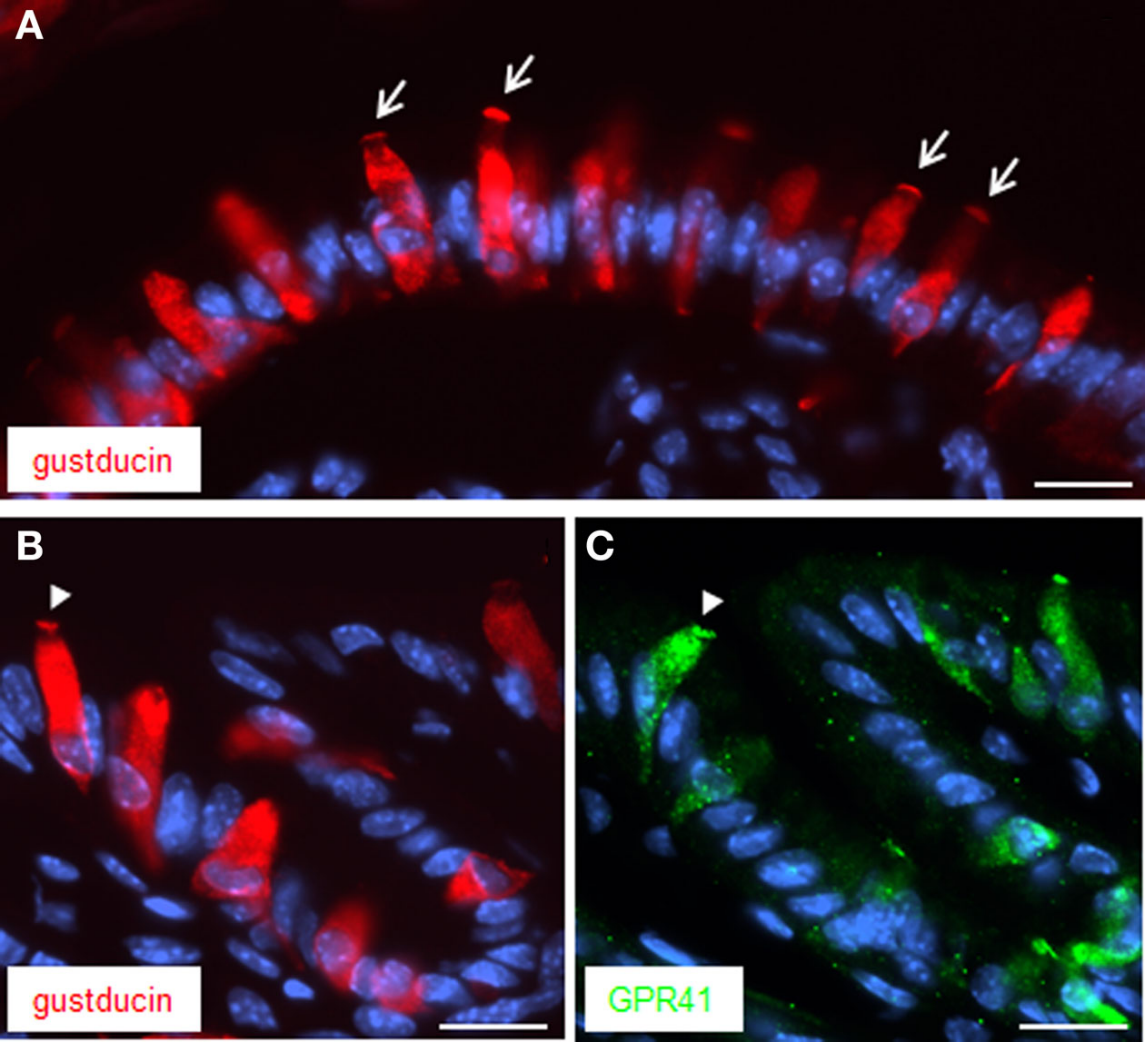
**Immunostaining for both α-gustducin and GPR41 in apical microvilli. (A)** Immunostaining for α-gustducin (*red*) resulted in strong staining of the apical cell pole, in appearance reminiscent to a dense brush of microvilli (denoted by *arrows*). **(B,C)** Treatment of consecutive tissue sections (6 μm) yielded similar staining patterns for α-gustducin **(B)** and GPR41 **(C)**. Strong similarities between both immunostainings got evident despite fainter fluorescence intensity for GPR41-antibody. Beyond the same population of cells, both antibodies appeared to mark the same subcellular compartment, the microvilli (designated by the *arrowhead*). Sections were counterstained with DAPI (*blue*). *Scale bars*: **(A–C)** = 20 μm.

Previous studies have demonstrated that the GPR41 receptor couples through proteins of the Gi/o family to signaling pathways of the cell (Le Poul et al., 2003). This result was recently substantiated by a study demonstrating co-expression and functional coupling of the GPR41 receptor with the G-protein α-gustducin (Li et al., 2013), which is a member of the Gi/o family (Hoon et al., 1995). It has previously been shown that brush cells at the “gastric groove” express α-gustducin (Höfer et al., 1996; Hass et al., 2007). Therefore, attempts were made to explore whether α-gustducin may be co-expressed with GPR41 in brush cells. The results of immunohistochemical studies are depicted in Figure 3A indicating several elongated α-gustducin positive cells. Some of the cells exhibit a particularly strong staining at their apical pole (indicated by *arrows*). This appearance is very reminiscent of the staining pattern obtained for GPR41. For technical reason (antibodies from the same species) double labeling experiments for α-gustducin and GPR41 were not possible. Therefore, thin consecutive tissue sections (6 μm) were used for immunohistochemical studies with antibodies for α-gustducin (Figure 3B) and for GPR41 (Figure 3C). The results clearly demonstrated the similarity of the staining patterns. Although the fluorescence intensity for GPR41 immunoreactivity was weaker than that for α-gustducin it is apparent that both antibodies labeled either the same, or largely overlapping, population of cells. Furthermore, as indicated by the *arrowheads* on the two consecutive tissue sections, cells with a robustly labeled apical region are visible in the same region.

**Figure 4:**
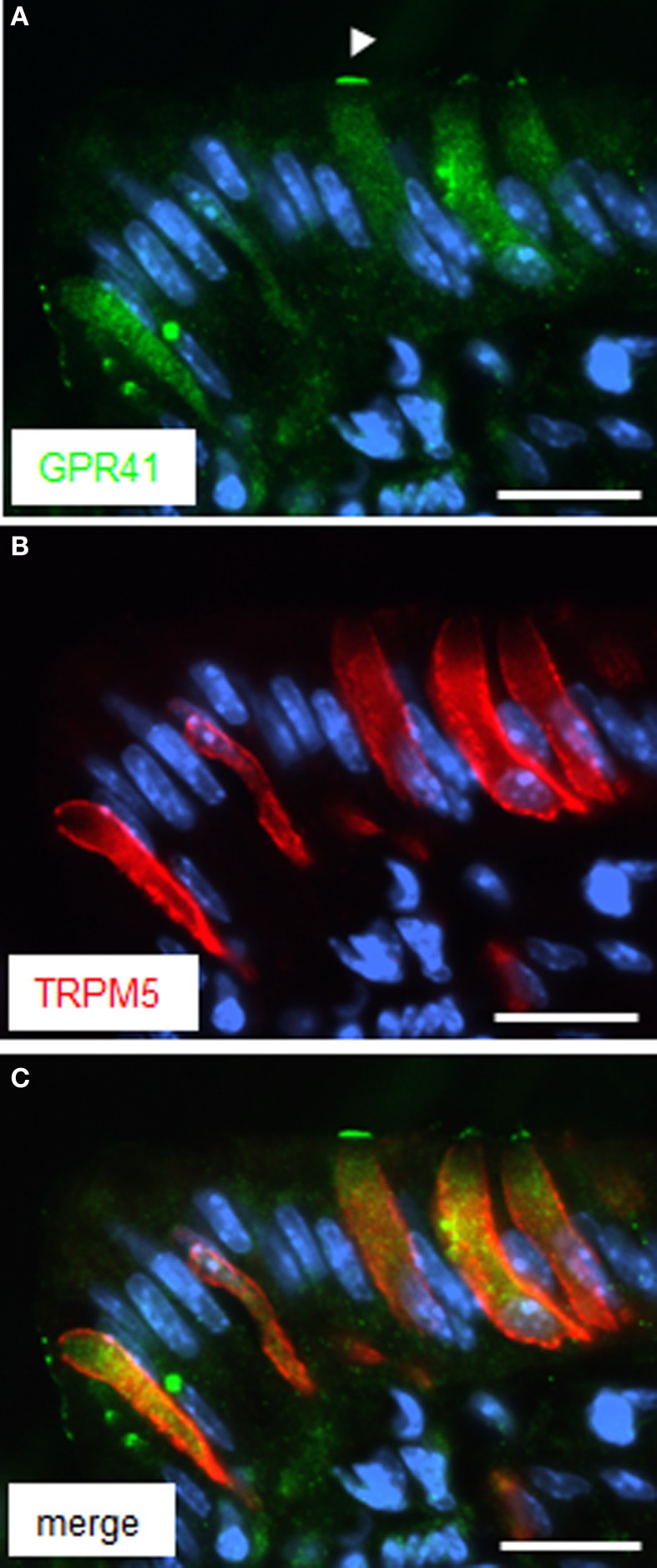
**Immunoreactivity for TRPM5 in GPR41 expressing cells. (A)** Several elongate cells were weakly stained by the GPR41-antibody *(green)*. The weaker staining intensity compared to Figures 1 and 2, respectively, is due to application of different GPR41 antibodies required for technical reasons. Stronger labeling, however was evident at the apical cell pole (*arrowhead*). **(B)** Immunohistochemical stainings with the TRPM5-antibody *(red)* resulted in a staining pattern similar for GPR41 in **(A)** revealing several elongate cells. **(C)** Overlay of **(A)** and **(B)** revealed an overlap of both immunostainings. Sections were counterstained with DAPI (*blue*). *Scale bars*: **(A–C)** = 20 μm.

Since brush cells express additional transduction elements of taste cells (Eberle et al., 2013), experiments were performed in order to explore whether GPR41 cells may express the cation channel TRPM5 (Hofmann et al., 2003). Double labeling experiments with antibodies for GPR41 and TRPM5 clearly demonstrated co-location, thus indicating co-expression of GPR41 and TRPM5 in brush cells at the “gastric groove” (Figure 4C). However, the TRPM5-antibody, like the CK18-antibody, led to a cytoplasmic labeling (Figure 4B), whereas the immunostaining for GPR41 was particularly strong for the apical cell pole, as marked by the *arrowhead* in Figure 4A. Whether the different staining pattern indicates a different distribution of TRPM5 compared to GPR41 or whether it is due to technical limitations of the respective antibodies is unclear.

**Figure 5:**
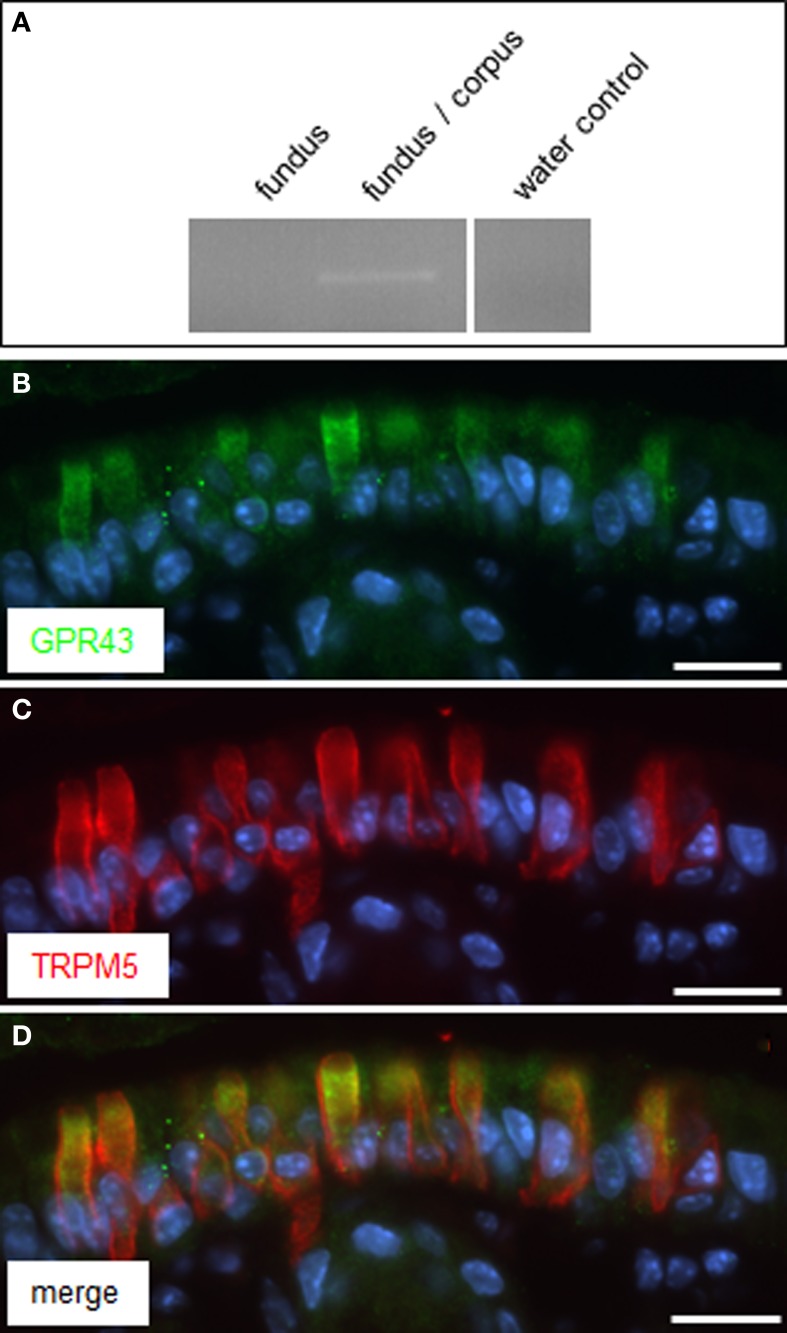
**GPR43 is expressed in TRPM5 positive cells at the “gastric groove.” (A)** RT-PCR approaches analyzing normalized cDNA of gastric tissue from fundus and the fundus/corpus boundary, respectively, were conducted with primer pairs specific for GPR43 (465 bp); for cDNA of the fundus/corpus transition zone a transcript of the expected size was obtained. Water control without template showed no amplicon. **(B,C)** Double-immunolabeling employing antibodies against GPR43 *(green)* and TRPM5 *(red)* on corpus tissue sections. The GPR43-antibody labeled numerous cells **(B)**. Co-staining with the TRPM5-antibody **(C)** revealed an overlap of both immunostainings **(D)**. Sections were counterstained with DAPI (*blue*). Scale *bars*: **(B–D)** = 20 μm.

Very recently it was reported that in enteroendocrine cells the receptor GPR41 may operate together with the related receptor GPR43, as co-sensors for short-chain fatty acids (Nøhr et al., 2013). The receptor type GPR43 is encoded at the same chromosomal locus as GPR41 (Sawzdargo et al., 1997) and also responds to the SCFAs propionate and butyrate (Brown et al., 2003; Le Poul et al., 2003; Nilsson et al., 2003). Therefore, it was analyzed whether brush cells at the “gastric groove” may also express GPR43. RT-PCR analysis resulted in a GPR43 specific amplification product for cDNA from tissue samples of the fundus/corpus transition region but not from fundus tissue (Figure 5A). Subsequent immunohistochemical analysis with specific antibodies led to labeling of cells at the “gastric groove.” Double-labeling experiments with antibodies for GPR43 (Figure 5B) and for TRPM5 (Figure 5C) revealed a complete overlap of labeled cells (Figure 5D). These results indicate that GPR43 receptors are in fact expressed in brush cells at the “limiting ridge.”

## Discussion

In the stomach of rodents, clusters of brush cells are arranged in a band-like manner between the proximal keratinized reservoir compartment and the more distal glandular part. Due to the expression of molecular elements typical of gustatory sensory cells, it has been suggested that these brush cells may have chemosensory capacity (Höfer et al., 1996; Hass et al., 2007, 2010; Eberle et al., 2013). However, since only few evidence for the expression of taste receptors in these cells exist (Hass et al., 2010; Janssen et al., 2012), it has been unclear which compounds may be sensed by them. In searching for candidate receptors, we have reasoned that the segregation of the cells at the border between the fundus and corpus compartments is suggestive of a capacity of these cells to detect chemical constituents in the fundus lumen. It has been shown previously that in the luminal content of the anterior part of the mouse stomach the pH value as well as the number of bacteria are relatively high (Smith, 1965; Kararli, 1995). While it is well known that a dense population of microbiota settles in the colon, it is generally assumed that, partly due to a strong acidic milieu, the stomach is not colonized by bacteria. However, the murine fundus accounts for approximately two-thirds of the whole stomach and is easily distensible, which allows prolonged storage of the ingested food (Gärtner, 2002). During the storage period, the resident microorganisms initiate fermentation processes in the predominantly anaerobic milieu, resulting in the generation of SCFAs (den Besten et al., 2013). The present results imply that the brush cells at the fundus border may sense these metabolites. Thus, the concentration of microbiota-derived short-chain fatty acids may be an important parameter in regulating processes such as the transfer of the ingesta from the storage compartment into the digestive compartment or secretory processes in the glandular digestive stomach regions. Monitoring the progress of ongoing fermentation processes in the reservoir compartment might also exert effects on the regulation of food ingestion, for example by affecting the release of the orexigenic hormone ghrelin. Interestingly, previous studies have demonstrated that ghrelin-producing cells are located in close vicinity to the brush cells (Hass et al., 2007). In view of these considerations, the present finding that receptors for the short-chain fatty acids are expressed in gastric brush cells at the “limiting ridge” is of particular importance. Both GPR41/FFAR3 and GPR43/FFAR2 have previously been shown to be responsive to SCFAs (Brown et al., 2003; Le Poul et al., 2003; Nilsson et al., 2003). Moreover, in a very recent study it has been suggested that the two receptor types may operate as “cosensors” for these metabolites (Nøhr et al., 2013). Propionate, acetate and butyrate represent the major SCFA types which are generated by fermentative processes (Cook and Sellin, 1998; Tremaroli and Bäckhed, 2012) and propionate appeared to be the most potent agonist for GPR41 as well as for GPR43 (Le Poul et al., 2003). Thus, the brush cells appear capable of sensing SCFAs and may use elements of the gustatory signaling cascade for the transduction process. Both receptor types couple through proteins of the Gi/o family to cellular signaling pathways (Le Poul et al., 2003) and the G-protein α-subunit gustducin, which is presumably co-expressed with GPR41 in the majority of brush cells, is member of the Gi/o family (Hoon et al., 1995). Furthermore, recent studies with α-gustducin knockout mice have provided experimental evidence for a functional coupling of GPR41 and GPR43 with α-gustducin (Li et al., 2013). The similar staining patterns for α-gustducin and GPR41 (Figures 3B,C) indicate that both proteins are located in the apical microvilli of the brush cells, a prerequisite for cooperation of both elements in the transduction cascade. In their studies, Le Poul et al. (2003) have shown that stimulation of GPR41 and GPR43 leads to the formation of 1,4,5-trisphosphate (IP_3_) and the activation of intracellular Ca^2+^ stores. Interestingly, brush cells at the “gastric groove” express phospholipase C beta2 (PLCβ2) which mediates the formation of IP_3_ (Eberle et al., 2013). Thus, brush cells at the “limiting ridge” comprise signaling elements, which may allow a responsiveness to SCFAs. The emerging picture suggests that in contrast to taste cells which assess the ingested food in the oral cavity, brush cells at the fundus border may use their chemosensory capacity to monitor the progress of fermentation in the reservoir compartment of the stomach. This information may be particularly relevant for the initiation and regulation of gastric processes, especially for the propulsion of chyme from the reservoir compartment to the glandular digestion compartment. In this context it is interesting to note that previous studies have shown that in the colon of rats SCFAs affect the motility and that this modulatory effect of SCFAs was apparently mediated by cholinergic and serotonergic mechanisms (Tazoe et al., 2008). Interestingly, for brush cells at the “gastric groove” it has previously been shown that they do have the potential for cholinergic transmission (Eberle et al., 2013), and moreover, multiple serotonergic cells are located in close vicinity of the brush cells, allowing paracrine interaction of both cell clusters (Hass et al., 2007). Consequently, it is conceivable that similar mechanisms may be active in the stomach.

### Conflict of interest statement

The authors declare that the research was conducted in the absence of any commercial or financial relationships that could be construed as a potential conflict of interest.

## Abbreviations

CK18: Cytokeratin 18
FFAR: free fatty acid receptor
SCFAs: short-chain fatty acids
TRPM5: Transient receptor potential cation channel, subfamily M, member 5.
